# Associations Between Heavy Episodic Drinking, Drinking While Gambling, and Risky Gambling

**DOI:** 10.1007/s10899-023-10235-w

**Published:** 2023-07-04

**Authors:** Koen Smit, Heng Jiang, Matthew Rockloff, Robin Room, Sarah MacLean, Anne-Marie Laslett

**Affiliations:** 1 Centre for Alcohol Policy Research, School of Psychology and Public Health, La Trobe University, NR1 Building, Melbourne, VIC 3086 Australia; 2 Melbourne School of Population and Global Health, University of Melbourne, Melbourne, Australia; 3 Department of Public Health, School of Psychology and Public Health, La Trobe University, La Trobe, Australia; 4 Experimental Gambling Research Laboratory, Central Queensland University, Queensland, Australia; 5 Centre for Social Research on Alcohol and Drugs, Department of Public Health Sciences, Stockholm University, Stockholm, 106 91 Sweden; 6 Social work and social policy, School of Allied Health, Human Services and Sport, La Trobe University, La Trobe, Australia; 7 National Drug Research Institute, Curtin University, Perth, WA Australia

**Keywords:** Risky gambling, Heavy episodic drinking, Alcohol use, Alcohol use while gambling, Public health

## Abstract

Introduction: Understanding how patterns of drinking are associated with risky gambling in Australia is needed to inform an effective approach to minimise harm. Methods: This cross-sectional questionnaire study reports on 2,704 subsampled participants who completed survey questions about their patterns of drinking. With logistic regressions, we examined whether frequency of heavy episodic drinking (HED) and alcohol use while gambling were associated with risky gambling while controlling for sociodemographic variables. Results: Occasional HED and monthly HED were associated with any gambling (versus no gambling), but frequent HED was not significantly associated with gambling. The opposite pattern was found when predicting risky gambling. Occasional HED (i.e. less than monthly) was not significantly associated, but a higher frequency of HED (at least weekly) was associated with a higher likelihood of risky gambling. Drinking alcohol while gambling was associated with risky gambling, over and above HED. The combination of HED and use of alcohol while gambling appeared to significantly increase the likelihood of risky gambling. Conclusions: The association of HED and alcohol use while gambling with risky gambling highlights the importance of preventing heavy alcohol use among gamblers. The links between these forms of drinking and risky gambling further suggests that individuals who engage in both activities are specifically prone to gambling harm. Policies should therefore discourage alcohol use while gambling for example by prohibiting serving alcohol at reduced prices or to gamblers who show signs of being affected by alcohol and informing individuals of the risks associated with alcohol use while gambling.

**Supplementary Information:**

The online version contains supplementary material available at 10.1007/s10899-023-10235-w.

## Introduction

Gambling can cause substantial harm to individuals, families and communities (Abbott, 2020; Langham et al., 2015). At population and individual levels, the recreational benefits of gambling may not compensate for these costs (Rockloff et al., 2019). Gambling is associated with serious health and social harms, such as psychological problems (e.g., feeling ashamed) and mental health disorders, addiction problems, financial insecurity (e.g., loss of savings) and decreased social well-being (e.g. decreased quality of relationships with significant others with less time spent) (Browne et al., 2018; Potenza et al., 2002; Price et al., 2021; Sulkunen et al., 2018).

The state of Victoria, Australia, is a state with a strong gambling culture (Delfabbro & King, 2012) and a less-than-ideal “responsible gambling” policy (Livingstone & Rintoul, 2020). About two-thirds of Victorian adults (69%) reported gambling within the last 12 months (Rockloff et al., 2020) and 1.2% reported problem gambling, as shown by the second national study of interactive gambling in Australia (Hing et al., 2021). Problem gambling was defined as experiencing adverse consequences because of gambling and having lost control of gambling behaviour. In Victoria, those who reported risky gambling (problem gambling and moderate risk gambling) reported higher levels of alcohol use both in general and while gambling (Victorian Populations Gambling and Health Study (2018-19). More clarity is needed on which specific patterns of alcohol use are associated with risky gambling behaviour. Therefore, the current study examined whether patterns of alcohol use, including heavy episodic drinking (HED), which might occur at any time rather than only while a person is gambling, and drinking whilst gambling, are associated with gambling and risky gambling.

Alcohol use is a major risk factor for acute and chronic negative health consequences such as injuries and cancer (Rehm et al., 2009), and also for harm to others around the drinker (Babor et al., 2023). Although alcohol use rates have declined, about 26% of Australians exceeded the drinking guidelines in 2020-21 (> 10 drinks last week and/or 5 or more drinks in one occasion), with men (vs. women), those born in Australia (vs. overseas) and those living in regional areas (vs. urban areas) being more likely to exceed these guidelines (ABS, 2020-21). Alcohol has a significant place in Australian culture, as well as in gambling situations, which evokes the question to what extent HED (drinking 6 or more drinks on one occasion (Frank et al., 2008) and alcohol use while gambling are related to gambling at all, and to risky gambling.

The Problem Behaviour Theory (PBT) posits that problem behaviours are not isolated and are interconnected because of similar underlying risk factors (Jessor, 1991; Jessor & Jessor, 1977). Indeed, prior evidence provides some support for associations between HED, drinking while gambling, and gambling problems. But the studies are based on various populations and show inconsistent results. In a sample of US college athletes, any kind of gambling deemed as ‘risky’ was associated with at-least-weekly HED (Huang et al., 2010). Conversely, another study found that moderate alcohol consumption *while* gambling was negatively associated with gambling duration, with a stronger effect shown on people deemed as having gambling problems (Markham et al., 2012). In line with the PBT, Kaltenegger and colleagues (Kaltenegger et al., 2019) found that gambling involvement and HED shared the underlying factors of psychological problems and impulsivity amongst Swedish adolescents, suggesting that certain individuals are at risk for either or both behaviours. As a basis for policies on alcohol availability at gambling sites, more evidence is needed, particularly in the Australian context, to add to previous findings and the theoretical underpinnings from the PBT.

The connection between gambling and alcohol use is made and encouraged at several levels. First, it is forced by government law in many places, including Australia. When governments in the later 20th century legalised electronic gambling machines, they were placed in areas where children’s access was restricted. Consequently, gambling machines are in taverns (including pubs, clubs, and casinos in Australia), thus forming an environmental connection between alcohol use and gambling. Second, many risky gamblers like to drink alcohol while gambling: 30% of moderate risk and problem gamblers in a large Victorian survey sample reported often or always drinking while gambling (Rockloff et al., 2020, p. 78). Third, casinos have long recognised that gamblers who are affected by alcohol are much more willing to take risks, and therefore facilitate alcohol availability and supply to the heavy gamblers which are a substantial part of their revenue (Rosengren, 2016). It is therefore useful to inform policy makers to identify patterns of alcohol use associated with risky gambling, particularly in the Australian context.

Alcohol use may impair judgement on the costs and benefits of gambling, and is shown to be associated with larger bets and faster loss of available funds (Cronce & Corbin, 2010). However, one study looked at alcohol use and promotion in sport bars and concluded that, although both behaviours occurred at the same time, no strong reciprocal relationship between alcohol and gambling was identified (Pennay et al., 2021). On the other hand, drinking was found to affect gambling losses on electronic gambling machines (EGMs). A placebo-controlled EGM experiment found that consuming one standard drink led to people persisting for twice as many gambling trials and being more likely to lose all their funds (Kyngdon & Dickerson, 1999). Therefore, alcohol use while gambling might not only be an important indicator of risky gambling, but also may be practiced by those who gamble less problematically. This point was also raised in a recent meta-analysis of 104 studies (Allami et al., 2021), showing an absence of association between alcohol use and risky gambling when controlling for a host of co-morbid risk factors. This may be because previous studies focused on general drinking patterns or failed to partial-out shared risks such as mental health problems.

Risky gambling and alcohol use do show several important similarities. There is evidence that alcohol use and gambling are complementary activities as they often co-occur, particularly within licensed gambling venues (French et al., 2008; Pennay et al., 2021). Risky gambling and alcohol use are commonly comorbid conditions in both clinical and non-clinical samples (Stewart & Kushner, 2005). Indeed, alcohol use and gambling were found to show similar dependency forming properties, with pathologic gamblers more at risk of alcohol abuse (Molde et al., 2009).

In various studies, HED is co-morbid with risky gambling. Those who generally drink more heavily and use alcohol as an intoxicant at more risk of becoming involved in risky gambling (Welte et al., 2004). Other studies show that those who problem gamble shared impairments in risky decision-making and cognitive impulsivity with individuals that are alcohol dependent (Kaltenegger et al., 2019; Lawrence et al., 2009). Additionally, HED is interesting to consider in association with risky gambling, as one might expect alcohol’s effects to become particularly salient after multiple drinks (Kyngdon & Dickerson, 1999; Welte et al., 2004). And both HED and drinking while gambling should be considered in the association with risky gambling, as both may contribute to harm caused by risky gambling in unique ways.

## Study Aims

Understanding how patterns of drinking may be associated with risky gambling in Australia is needed to inform an effective approach to minimise harm. Therefore, this study focused on two different indicators of alcohol use – HED and drinking alcohol while gambling -- as predictors of risky gambling. First, we examined the association of HED and alcohol use while gambling with gambling risk categories and sociodemographic groups. Second, we assessed associations of the alcohol use indicators with gambling participation (any gambling vs. no gambling) and risky gambling (risky gambling vs. some gambling). It was hypothesised that HED and alcohol use while gambling independently predicted whether a person was classified as an at-risk gambler. Third, we examined the interaction between alcohol use indicators and risky gambling. In accordance with the Problem Behaviour Theory, we expected that the combination of HED and alcohol use while gambling would have an exacerbating effect on a gambler’s risk classification.

## Methods

### Study Design and Procedure

The current study involved 10,638 computer-assisted telephone interviews (CATI) (Rockloff et al., 2020). The questionnaire was designed by Central Queensland University’s Experimental Gambling Research Laboratory. Most participants received a shortened version of the survey (core data items), whereas 2,704 participants were subsampled and were asked additional survey questions – such as about their patterns of drinking.

To maximise information from those who are involved in risky gambling, the long version of the questionnaire was administered to all at-risk gamblers, i.e. low risk, moderate risk and problem gamblers according to the Problem Gambling Severity Index (PGSI). Additionally, the long version was administered to randomly selected non-gamblers (32.0%) and non-problem gamblers (11.5%). Sub-sample population weights were used to scale prevalence figures to the adult population of Victoria, Australia, at the time of the survey (N = 4,919,289).

This study followed the STROBE guidelines (Von Elm et al., 2007). Ethics approval was obtained from the Human Research Ethics Committee at Central Queensland University (Application Reference 21,134).

### Measures

*Gambling participation.* Gambling activity was documented as a binary outcome and assessed as either present or absent in the previous 12 months.

*Gambling risk profile*. To assess the prevalence of problem gambling and associated harms, all respondents who had participated in at least one gambling activity in the last twelve months were asked the nine-item Problem Gambling Severity Index (PGSI, (Ferris & Wynne, 2001). The PGSI is a subset of questions drawn from the larger Canadian Problem Gambling Index, which is a standardised screening tool that is used widely in international and Australian gambling prevalence surveys. The PGSI contains response categories between 0 (*never*) and 3 (*almost always*). Sum scores are used to form a risk category score: (0) non-problem gambler, (1–2) low risk gambler, (3–7) moderate risk gambler, or (8–27) problem gambler. All participants were included and their PGSI scores were recoded to (0) non-gambler if no gambling was indicated in the last year, (1) non-problem or low risk gambler, and (2) moderate risk or problem gambler. Although there is some overlap in gambling behaviours, it is typical to report moderate-risk and problem gambling separately. However, the low-prevalence of the moderate and problem-gambling risk categories necessitated combining these two categories as risky gambling for the purposes of our analyses.

*Frequency of alcohol use while gambling*. Participants responded to the question “During the past 12 months, how often did you drink alcohol while gambling?” on a 5-point Likert scale from 1 (never) to 5 (always). Those who did not know or refused to answer, were coded as missing (n = 4, 0.2%).

*Heavy episodic drinking (HED).* Respondents who reported drinking in the past 12 months (including gamblers and non-gamblers) were asked the first three Alcohol Use Disorders Identification Test (AUDIT-C) questions. The AUDIT is a brief screening test of 10 questions to help identify risky drinking and harmful alcohol use (Frank et al., 2008). For the current analysis, we extracted the frequency of consuming six or more drinks (i.e. 60 gm of pure alcohol) on one occasion [in the previous 12 months]: never, less than monthly, monthly, and weekly, or daily (HED). This was dichotomized in some analyses (0 = no vs. 1 = any HED). We were specifically interested in alcohol’s effects among individuals who are more likely to be intoxicated, occasionally or even frequently.

*Demographics.* The participants’ gender, age, region in Victoria (Melbourne vs. outside of the capital), family background (as indicated by English vs. another language used at home), and yearly personal income were recorded. Further specifications of response categories are included in Table 1.

**Table 1:** Weighted percentages of non-gamblers, low-risk gamblers, and high-risk gamblers by sociodemographic characteristics

		All Victorians (N = 2,672)				Victorians who gambled in last year (n = 1,720)	
		%	%	%			
	N	Non-gambler	No and low risk gambler	Moderate and problem gambler		No and low risk gambler	Moderate and problem gambler
All Victorian adults	2672	30.9%	65.9%	3.1%		95.5%	4.5%
Gender							
Male	1,345	30.1%	65.6%	4.3%		93.8%	6.2%
Female	1,327	31.8%	66.3%	1.9%		97.2%	2.8%
Age							
18–34	617	43.2%	52.7%	4.1%		92.3%	7.2%
35–49	500	26.3%	70.6%	3.1%		95.8%	4.2%
50–64	692	23.3%	73.9%	2.8%		96.3%	3.7%
65+	863	26.9%	71.2%	2.0%		97.3%	2.7%
Region in Victoria							
Melbourne	2031	32.5%	64.3%	3.1%		95.3%	4.7%
Regional	641	25.6%	71.4%	3.0%		96.0%	4.0%
First language at home							
English	2104	26.1%	71.0%	2.9%		96.0%	4.0%
Other	565	47.9%	48.4%	3.7%		92.9%	7.1%
Personal Income, per year							
Nil - $41,599	1,070	36.9%	59.5%	3.5%		94,4%	5.6%
$41,600 - $77,999	485	31.1%	64.9%	4.0%		94.2%	5.8%
$78,000 or more	495	20.8%	76.3%	2.8%		96.4%	3.6%
Missing	622	32.5%	65.5%	2.0%		97.0%	3.0%
HED							
Never	1669	36.3%	61.7%	2.1%		96.7%	3.3%
Less than monthly	560	26.2%	71.3%	2.5%		96.6%	3.4%
Monthly	264	18.0%	76.7%	5.3%		93.6%	6.4%
Weekly or daily	179	25.1%	64.4%	10.4%		86.0%	13.9%
Drinking alcohol while gambling^a^							
Never	1,076	-	-	-		97.3%	2.7%
Sometimes	429	-	-	-		93.6%	6.4%
Often/always	211	-	-	-		87.2%	12.8%

Note: ^a^ Non-gamblers are excluded

### Analyses

Descriptive statistics were used to examine the distribution of the gambling risk group categories (PGSI) by sociodemographics, geographical location, and by drinking patterns. Weighted percentages were shown for the full population of the study and for those who indicated gambling in the last year. Second, logistic regressions were used to test bivariable and multivariable associations between HED (6 + drinks on one occasion) with the risk for any gambling (any gambling vs. no gambling) while controlling for sociodemographic factors. Weighted data, with the n set to the sample size of 2,704, were used to adjust the findings to the Victorian population (Rockloff et al., 2020), and the estimates are presented with confidence intervals. Among those who indicated gambling in the last year, logistic regressions tested the associations of HED and risky gambling vs. any gambling (i.e. moderate and problem gambling vs. no and low-risk gambling). First, we used HED as predictor (model 1) and in a separate analysis (model 2), we added alcohol use while gambling to investigate whether this would make a difference. Subsequently, we used bivariable and multivariable logistic regressions to test the interaction of HED and alcohol use while gambling with risky gambling (vs. any gambling). Since multiple cell sizes were small (n < 30), we dichotomized HED (0 = No HED, 1 = any HED), and alcohol use while gambling (0 = no alcohol use while gambling, 1 = any alcohol while gambling) to determine whether the combination of both drinking patterns affected the likelihood of risky gambling. All analyses were performed using Stata 17 (StataCorp, 2021).

## Results

### Characteristics of the Sample

Characteristics of the sample, including gambling prevalence estimates by sociodemographic characteristics for the full population and those who gambled during the last year, are reported in Table 1. About 3.1% of Victorians were estimated to be involved in risky gambling, whereas about two thirds of Victorians were low-risk gamblers. Among those who gamble at all, about 4.5% were classified as a risky gambler. We found that relatively more men than women (4.3% vs. 1.9%), and a higher percentage of those in the younger age groups were risky gamblers. Relatively more adults indicated being a risky gambler if they engaged in frequent HED (weekly or daily), with about one third of those who engage in regular HED also being a risky gambler. Similar elevations in rates of high-risk gambling were found among those who drink sometimes and more frequently (often/always) during gambling.

[Table 1 about here]

### Predictors of Gambling (vs. no Gambling)

Table 2 shows estimates of whether frequency of HED (6 + drinks) and sociodemographic variables are associated with the odds of *any* gambling vs. no gambling. Bivariable logistic regression analyses show that all categories of people with HED are more likely to engage in any gambling form, as compared to the reference group (no HED). This association remained true when controlling for sociodemographics in the multivariable analysis. No significant differences were found between men and women. Respondents in the older age groups (compared to 18–34 years old) and who were living in regional Victoria were at higher odds of gambling. However, the latter association disappeared in the multivariable analysis. Those who spoke English at home were about two times more likely to be gamblers than those speaking another language at home. Lastly, it appeared that those in the highest income group, were about half as likely as those with lower income to be a gambler.

**Table 2:** Unadjusted and adjusted odds of any gambling (versus none) by alcohol use and sociodemographic characteristics

None vs. Any gambling (weighted)		Bivariable logistic regression		Multivariable logistic regression			
	N	Unadjusted OR		Adjusted OR	95% CI		*p*
All Victorian adults	2,672						
**Gender**							
Male	1,345	1.00					
Female	1,327	0.92		1.10	0.86	1.42	0.445
**Age**							
18–34	617	1.00					
35–49	500	2.13***		2.07***	1.42	3.02	< 0.001
50–64	692	2.50***		2.52***	1.78	3.57	< 0.001
65+	863	2.07***		2.55***	1.78	3.66	< 0.001
**Region in Victoria**							
Melbourne	2031	1.00					
Regional	641	1.40*		1.07	0.83	1.51	0.439
**First language at home**							
English	2104	1.00					
Other	565	0.38***		0.53***	0.39	0.73	< 0.001
**Personal Income, per year**							
Nil - $41,599	1,070	1.00					
$41,600 - $77,999	485	1.29		1.24	0.88	1.77	0.222
$78,000 or more	495	2.22***		1.81**	1.25	2.61	0.002
Missing	622	1.22		1.08	0.77	1.5	0.667
**HED**							
Never	1,669	1.00					
Less than monthly	560	1.60**		1.74**	1.26	2.43	0.001
Monthly	264	2.60***		3.13***	1.94	5.07	< 0.001
Weekly or daily	179	1.70*		1.67	0.98	2.84	0.060

*Note.* *p < .05, ** *p* < .01, ***p < .001; Exact p-values are provided for the multivariable analyses.

### Predictors of Risky Gambling (vs. any Gambling)

Table 3 presents information on bivariable and multivariable logistic regressions of alcohol use with *risky* gambling (i.e. moderate-risk, and problem-gambling) vs. no-risk gambling. The results show that respondents who were males, from a younger age group, spoke a language other than English at home, and had lower personal income were more likely to report risky gambling in the past 12 months. Those with a drinking pattern of weekly HED (vs. less frequent or no HED) and those reporting alcohol use during gambling (vs. no alcohol while gambling) were at higher odds of being risky gamblers. No significant association was found for regional versus metropolitan Victorians.

**Table 3:** Unadjusted and adjusted odds of risky gambling, among those gambling at all, by alcohol use and sociodemographic characteristics

Some vs. risky gambling (weighted)	Bivariable logistic regression			Multivariable logistic regression				
				Model 1	Model 2			
	N	Unadjusted OR		Adjusted OR	Adjusted OR	95% CI		*p*
All Victorian adults	1720							
**Gender**								
Male	902	1.00		1.00	1.00			
Female	818	0.66***		0.51**	0.52**	0.35	0.79	0.002
**Age**								
18–34	343	1.00		1.00	1.00			
35–49	347	0.57**		0.78	0.90	0.53	1.50	0.677
50–64	472	0.49**		0.66	0.69	0.41	1.16	0.164
65+	558	0.36***		0.48**	0.56*	0.33	0.96	0.033
**Region in Victoria**								
Melbourne	1,289	1.00		1.00	1.00			
Regional	431	0.85		0.80	0.74	0.45	1.23	0.259
**First language at home**								
English	1,407	1.00		1.00	1.00			
Other	311	1.84**		1.73*	1.91*	1.16	3.12	0.011
**Personal Income, per year**								
Nil - $41,599	668	1.00		1.00	1.00			
$41,600 - $77,999	331	1.04		0.80	0.75	0.46	1.26	0.259
$78,000 or more	353	0.62*		0.47**	0.42**	0.24	0.72	0.002
Missing	368	0.52**		0.46**	0.44**	0.25	0.76	0.003
**HED**								
Never	974	1.00		1.00	1.00			
Less than monthly	397	1.04		0.85	0.69	0.42	1.12	0.132
Monthly	211	2.03**		1.69	0.90	0.49	1.67	0.738
Weekly or daily	138	4.80***		4.00***	2.25**	1.22	4.15	0.009
**Drinking alcohol while gambling**								
Never	1,076	1.00		--	1.00			
Sometimes	429	2.49***		--	2.49***	1.56	3.96	< 0.001
Often/always	211	5.32***		--	4.29***	2.53	7.26	< 0.001

*Note.* *p < .05, ** *p* < .01, ***p < .001; Exact p-values are provided for the multivariable analyses; ^a^Model 1 includes HED only, model 2 includes HED and alcohol use while gambling.

In the next analysis, the interaction between HED and alcohol use while gambling was significantly associated with risky gambling. Engaging in both HED and alcohol use while gambling was associated with a higher likelihood of risky gambling (vs. any gambling), while controlling for sociodemographic indicators. This result suggests that those individuals who engage in HED *and* use alcohol while gambling are about three times more likely to be in the risky gambling group compared with those had no HED and who did not drink alcohol while gambling. Those who engaged in alcohol use while gambling, but no HED, also showed higher odds for risky gambling. However, engaging in HED in isolation was not significantly associated with risky gambling. The proportions of those who HED and drink while gambling (or both) are shown in Table 4. While about one in three risky gamblers reported neither of the drinking patterns, about 40% of risky gamblers reported both HED and drinking while gambling.

**Table 4:** Interaction of heavy episodic drinking (HED) and alcohol while gambling with risky gambling (vs. any gambling)

	HED	Alcohol use while gambling	Low risk gamblers	High risk gamblers	OR of interactions [95% CI]	*p*
			N = 1,431	N = 285	N = 1716	
1	No	No	47.3%	35.1%	1.00	--
2	Yes	No	18.9%	11.6%	0.65 [0.36–1.16]	0.145
3	No	Yes	11.4%	13.0%	2.07 [1.14–3.73]	0.016
4	Yes	Yes	22.7%	40.4%	3.16 [1.98–5.41]	0.035

*Note.* Rates of risky gambling among individuals grouped based on Heavy Episodic Drinking (HED: yes/no) and alcohol use while gambling (yes/no); Sociodemographic variables (gender, age, region in Victoria, first language at home and personal income) were controlled in the interaction analysis.

## Discussion

This study aimed to investigate the association of heavy episodic drinking (HED) and alcohol use while gambling with risky gambling. The results suggest that occasional HED (occasional or monthly) was associated with any gambling (versus no gambling), but frequent HED was not associated with engagement in gambling. The opposite pattern was found when predicting just risky gambling. Occasional HED (i.e. less than monthly) was not significantly associated with risky gambling, but a higher frequency of HED (particularly at least weekly) was associated with a higher likelihood of risky gambling (see results in Table 3). For risky gambling, drinking alcohol *while* gambling remained a significant predictor over and above HED. Moreover, the combination of HED and use of alcohol while gambling appeared to significantly increase the likelihood of risky gambling. The results confirm the hypothesis advanced in the introduction, i.e. when both drinking patterns occur, individuals are more likely to be risky gamblers.

These findings particularly suggest that reducing the frequency of episodic drinking and discouraging people from drinking alcohol during gambling could reduce risky gambling and related harms. The results further suggest, in accordance with Problem Behaviour Theory (Jessor, 1991; Jessor & Jessor, 1977), that alcohol use and (risky) gambling co-occur within individuals. One possible explanation is that individuals may have personalities with risk-taking characteristics, including the trait of impulsivity (Ioannidis et al., 2019), which would be an underlying factor of both drinking and gambling. On the other hand, those who consume several alcoholic beverages may engage risky practice due to intoxication (Kyngdon & Dickerson, 1999) that they normally would not. Neither scenario can be ruled out with the current dataset and therefore future studies should investigate whether risk-taking personality types explain the association between alcohol use and (risky) gambling and associated harm.

As noted in the introduction, there is some mixed evidence regarding drinking while gambling and harmful gambling. Kyngdon and Dickerson (1999) found experimentally that the ingestion of alcohol caused people to gamble longer and lose more money on an EGM (Kyngdon & Dickerson, 1999). However, conversely, Markham et al. (2012) found that moderate alcohol consumption while gambling was negatively associated with gambling duration in a single session (Markham et al., 2012). Our results lend support to the findings of Kyngdon and Dickerson (1999). Importantly, however, both previous studies relate to a single episode of gambling (Kyngdon & Dickerson, 1999; Markham et al., 2012), whereas our current results reflect the more general association across gambling episodes.

### Limitations and Future Studies

The current study draws from a recent and large Victorian gambling study dataset which allowed us to examine the association of alcohol use and risky gambling in a population where alcohol use and gambling are culturally embedded and seemingly co-dependent (Delfabbro & King, 2012). However, several limitations must be acknowledged. Although we have information on HED and alcohol use while gambling and we statistically control for each in multivariate analyses, the current data did not allow us to examine to what extent the HED takes place *while* gambling.

Future studies should address the contexts and mechanisms behind how heavy episodic drinking *while* gambling affects risky gambling. Since we used cross-sectional data, no temporal inferences can be made. Information on longitudinal associations were therefore missing, as was data on whether HED precedes risky gambling, or the other way around. Lastly, the current study focused on gambling in general. Greater insight into associations of alcohol use with various gambling products, particularly those that are often available in licensed settings such as electronic gambling machines, would additionally assist the development and targeting of strategies for prevention to reduce risky gambling and associated gambling harm. Qualitative methods could provide a deeper understanding of the relationship between gambling and drinking patterns, which could inform targeted interventions to improve people’s health and wellbeing.

### Implications

The study provides insights for policy makers and practitioners to consider. It shows that there is a higher risk of risky gambling related to various drinking patterns, and therefore, reducing alcohol consumption among people who gamble could decrease the risk of gambling problems. The results also suggest that drinking alcohol while gambling should be reduced. Moreover, the combination of HED and use of alcohol while gambling is a particular risk factor for gambling problems. The strong link between HED and alcohol use while gambling with gambling risk suggests that individuals who engage in both forms of drinking may have underlying vulnerabilities or that the combined use of these products can amplify the harm.

Specific recommendations include prohibiting gambling facilities, such as casinos and hotels, from serving alcohol for free or at greatly subsidised prices. Liquor licensing enforcement at premises where gambling and alcohol products are both provided should ensure that gamblers who show signs of being affected, e.g., slurred speech or awkward movement, should not be served alcohol. Furthermore, individuals who gamble should be informed of the risks associated with drinking while gambling. For instance, warnings or guidelines about these risks could be posted in gambling establishments, or disseminated widely via media, advertising, and marketing campaigns. Cross-collaborations and referral pathways between gambling and addiction treatment services should also be strengthened to reduce alcohol-influenced risky gambling and its associated harms.

### Electronic Supplementary Material

Below is the link to the electronic supplementary material.

Supplementary Material 1

## Data Availability

Restrictions apply to the availability of these data, which were used under license for this study. The data that support the findings of this study are available from Central Queensland University (CQUniversity), Australia.
