# Supplementary material for: Associations Between Heavy Episodic Drinking, Drinking While Gambling, and Risky Gambling
Source: J Gambl Stud. 2023 Jul 4;39(4):1597–610. doi: 10.1007/s10899-023-10235-w (PMC10628030; doi:10.1007/s10899-023-10235-w)
Supplement: Supplementary file 1 — Supplementary Material 1 [file 10899_2023_10235_MOESM1_ESM.pdf]

Dr. Koen Smit

Centre for Alcohol Policy Research,
Building NR 1, La Trobe University,
Bundoora, 3086, VIC (Australia)

☏ +61 490345737 (AUS); +31 6 25115212 (NL)
🖂 k.smit@latrobe.edu.au

Dear Editor,

Thank you for the opportunity to revise the manuscript entitled "*Associations between heavy episodic drinking, drinking while gambling, and risky gambling*".

In response to the reviewer's suggestion, we integrated the Problem Behaviour Theory as a guiding theory. We remain at your disposal should you have any further queries.

Thank you for considering publication of our work in *Journal of Gambling Studies.*

Warm regards, on behalf of all authors,
Koen Smit
